# Effects of 6-Month Multimodal Physical Exercise Program on Bone Mineral Density, Fall Risk, Balance, and Gait in Patients with Alzheimer’s Disease: A Controlled Clinical Trial

**DOI:** 10.3390/brainsci11010063

**Published:** 2021-01-06

**Authors:** A. Silvia Puente-González, M. Carmen Sánchez-Sánchez, Eduardo J. Fernández-Rodríguez, J. Elicio Hernández-Xumet, Fausto J. Barbero-Iglesias, Roberto Méndez-Sánchez

**Affiliations:** 1 Department of Nursing and Physiotherapy, Universidad de Salamanca, 37007 Salamanca, Spain; silviapugo@usal.es (A.S.P.-G.); csanchez@usal.es (M.C.S.-S.); edujfr@usal.es (E.J.F.-R.); fausbar@usal.es (F.J.B.-I.); 2 Instituto de Investigación Biomédica de Salamanca (IBSAL), 37007 Salamanca, Spain; 3 Department of Physical Medicine and Pharmacology, University of La Laguna, 38200 Santa Cruz de Tenerife, Spain; jhernanx@ull.es

**Keywords:** Alzheimer’s disease, physical exercise, multimodal exercise, multitarget-directed intervention, bone density, physical function, gait, balance, falls, cognitive impairment

## Abstract

We aimed to determine the short- and medium-term effects of a multimodal physical exercise program (MPEP) on bone health status, fall risk, balance, and gait in patients with Alzheimer’s disease. A single-blinded, controlled clinical trial was performed where 72 subjects were allocated in a 3:1 ratio to an intervention group (IG; *n* = 53) and control group (CG; *n* = 19), where the IG’s subjects were admitted to live in a State Reference Center of Alzheimer’s disease, which offers the targeted exercise program, while the CG’s subjects resided in independent living. A multidisciplinary health team assessed all patients before allocation, and dependent outcomes were again assessed at one, three, and six months. During the study, falls were recorded, and in all evaluations, bone mineral density was measured using a calcaneal quantitative ultrasound densitometer; balance and gait were measured using the performance-oriented mobility assessment (POMA), the timed up and go test (TUG), the one-leg balance test (OLB), and the functional reach test (FR). There were no differences between groups at baseline for all outcome measures. The prevalence of falls was significantly lower in the IG (15.09%) than in the CG (42.11%) (χ^2^ = 5.904; *p* = 0.015). We also found that there was a significant time*group interaction, with a post hoc Šidák test finding significant differences of improved physical function, especially in gait, for the IG, as assessed by POMA-Total, POMA-Gait, and TUG with a large effect size (ƞ^2^p = 0.185–0.201). In balance, we found significant differences between groups, regardless of time, and a medium effect size as assessed by POMA-Balance and the OLB (ƞ^2^p = 0.091–0.104). Clinically relevant effects were observed, although without significant differences in bone health, with a slowing of bone loss. These results show that a multimodal physical exercise program reduces fall risk and produces an improvement in gait, balance, and bone mineral density in the short and medium term in institutionalized patients with Alzheimer’s disease.

## 1. Introduction

The aging of the population is a process that is affecting the provision of health and social care from a public health perspective, and the situation is expected to worsen in the future. Increased life expectancy has led to a rise in the prevalence of chronic diseases, such as Alzheimer’s disease (AD), and increased morbidity at older ages [1,2]. It is estimated that by 2050, the number of patients with AD worldwide will increase approximately 300%, which will incur high costs globally [3,4].

Frailty is an age-related state characterized by a progression toward functional decline and increased risk of poor clinical outcomes [5,6]. Frailty is therefore often defined as a risk factor that predisposes a person to disability and dependence on others for activities of daily living (ADLs), leading to hospitalization and institutionalization [7]. However, consensus is lacking on some aspects of frailty, such as on a definition of frailty, and validation for different economic and clinical contexts. This is required to optimize frailty assessments and subsequent treatment choices and care planning [8].

Physical frailty and cognitive impairment are related, sharing physiopathological bases and some outcomes, such as gait disturbances, falls, fractures, disability, and even mortality [9,10]. During aging, changes in the sensorimotor and vestibular systems influence mobility and balance, increasing the risk of falls [11,12]. This also occurs in patients with AD, in whom cognitive impairment is associated with gait and balance disorders [13,14,15], which influences executive functions, attention, and visuospatial perception, increasing the risk of falls [16,17,18,19]. Patients over 65 years of age with dementia are at twice the risk of falling compared with elderly people without dementia [12,20,21,22].

Falls are directly related to bone fractures, causing significant complications, such as increased fear of falling, loss of autonomy, risk of disability, decreased quality of life, and anticipated mortality in elderly patients [23,24,25]. In addition, low bone mass conditions weaken the skeleton and increase the likelihood of bone fractures [23,24]. Cognitive impairment is also significantly associated with low bone mineral density (BMD) [26,27]. Bone fragility is a clinical comorbidity in AD [26], and AD patients have over twice the risk (2.5–2.7-fold) of experiencing clinically low BMD and hip fracture than cognitively healthy adults [28,29]. Chang et al. reported that older women in Asian populations with reduced BMD diagnosed with osteoporosis or osteoporotic fractures are at increased risk of cognitive impairment [30].

Therefore, the importance of gait and balance disturbances in the risk of falling must be considered. Gait alterations appear in 50% of AD patients three years after AD diagnosis; amongst these, 33% lose their capacity to walk. The prevalence of gait and balance alterations ranges from 9% to 52% depending upon the tool used for assessment [14,22,31]. Many tests can be used to assess gait and balance in the elderly, those with cognitive deficit, or AD patients, including: Tinetti’s performance-oriented mobility assessment (POMA-T), the timed up and go test (TUG), the one-leg balance test (OLB), and the functional reach test (FR) [11,13,22,31,32,33,34,35]. For this reason, they were selected for use in our study to assess balance and gait as indicators of physical frailty along with the risk of falls [10].

Currently, both pharmacological and non-pharmacological treatments are often applied for AD, other dementias, or cognitive impairment in older people. At present, only a few symptom-targeted pharmacological treatments are available, which provide modest improvements in patients’ livelihoods, and usually with side effects that may include nausea, dizziness, and weight loss [36,37,38,39]. For this reason, research on non-pharmacological treatments, which can be well implemented in the daily routine of patients, has increased in recent years. Physical activity is a viable and promising low-cost, low-risk, and widely available option, and is already known for its effects in reducing health risks in AD and other dementias, as well as in aging and other pathologies such as cardiovascular diseases, type 2 diabetes, arthritis, depression, cancer, or osteoporosis [36,40,41,42,43]. Physical activity or exercise is known to improve the cognition of elderly people with AD [16,42,44], and produces other beneficial effects, including improving physical function, and increasing autonomy and quality of life in patients with dementia [2,16,31,32].

The American College of Sports Medicine recommends 150 min of moderate or 75 min of vigorous physical activity per week, preferably divided into three to five sessions, and muscle-strengthening activities two or more days per week, with a recommendation of a 3:2 ratio of aerobic and strength exercise per week [36,45,46]. Multidomain supervised intervention is feasible and safe with low dropout rates and high intervention adherence [16].

Multimodal or multi-component exercise programs are possibly the most efficient non-pharmacological intervention in older people [10,16,47,48,49,50]. Different exercises, such as strength training, aerobic training, balance training, and flexibility training, can be combined, providing marked improvements in functional ability [2,10], and with good results if cognitive exercises are added as a dual-task intervention [16]. Dual-task interventions produce good results not only in patients with cognitive impairment, but also in patients with neurologic disorders, chronic pain, and the elderly in different conditions [51,52,53,54]. Physical exercise is one of the most important interventions to improve the functional capacity of frail elderly people, and according to current evidence, muscle strengthening should be at the forefront of treatment [10,55].

Exercise can be considered a useful tool to improve functional capacities associated with AD, such as mobility, gait, balance, strength, and executive functions, and thus decrease falls [2,10,15,44,47,48,49,56,57,58,59]. Exercise or planned physical activity has beneficial effects on regulating bone metabolism and maintaining optimal bone health [43,60,61,62,63]. Based on the evidence, a higher muscle mass and mechanical stress on bones result in increased or prevented BMD loss [43,64,65]. Therefore, regular exercise with moderate intensity decreases bone resorption and increases bone mass in both healthy and pathological subjects [66,67].

In this clinical trial, a multimodal physical exercise program (MPEP) was designed in institutionalized elderly people diagnosed with AD through a group intervention. The main objective of this trial was to improve bone health status, gait, and balance, as indicators of physical frailty, to decrease the risk of falls and fractures in people with AD temporarily institutionalized to the State Reference Center for people with Alzheimer’s disease and other dementias (SRC-Alzheimer). The aim of this study was to evaluate the short- and medium-term effects, over six months, of an MPEP on BMD, fall risk, balance, and gait using a controlled clinical trial in patients with AD.

## 2. Materials and Methods

### 2.1. Study Design

This study was a single blinded, controlled clinical trial. The protocol of the clinical trial received approval from the Ethics Committee of University of Salamanca (200900044302), and was conducted in accordance with the Declaration of Helsinki. The clinical trial was registered in the Australian New Zealand Clinical Trials Registry (ACTRN12618000872235) and conformed to the CONSORT 2010 Statement (Consolidated Standards of Reporting Trials) [68]. 

In this study, patients were consecutively recruited among the requests for admission to the SRC-Alzheimer (Salamanca, Spain) and the trial was conducted to evaluate the short- and medium-term effects of the intervention. The experimental part of the study was conducted at the SRC-Alzheimer over 3.5 years, with subsequent treatment, analysis, and interpretation of the data. The participants in the intervention group received an MPEP over 6 months, whereas participants in the control group received usual care and maintained their physical activity without systematic and supervised exercises during the same period. During the 6 months of the study intervention, there were four evaluations in both groups, at baseline, and at 1, 3, and 6 months after the start of the intervention. 

### 2.2. Sample Size Calculation

The sample size was based on a 1-month follow-up pilot study using the free software GRANMO sample size calculator (version 7.12, IMIM, Barcelona, Spain), considering POMA-T as the primary outcome. In this pilot study, the POMA-T was modified by 1.7 points with a standard deviation of 2 points. After this, we started the recruitment for this trial considering a ratio of 3:1 of the intervention to the control group. Accepting an α risk of 0.05 and a β risk of 0.2 in a two-sided test, 52 subjects were deemed to be necessary in first group and 17 in the second to recognize a difference greater than or equal to 1.7 units as statistically significant. The common standard deviation was assumed to be 2.0. We anticipated a drop-out rate of 15%. Due to the difficulties in recruiting non-institutionalized participants diagnosed with Alzheimer’s disease who not participate in an exercise program, we decided to consider a 3:1 ratio between the two groups.

### 2.3. Participants and Recruitment

**Figure 1:**
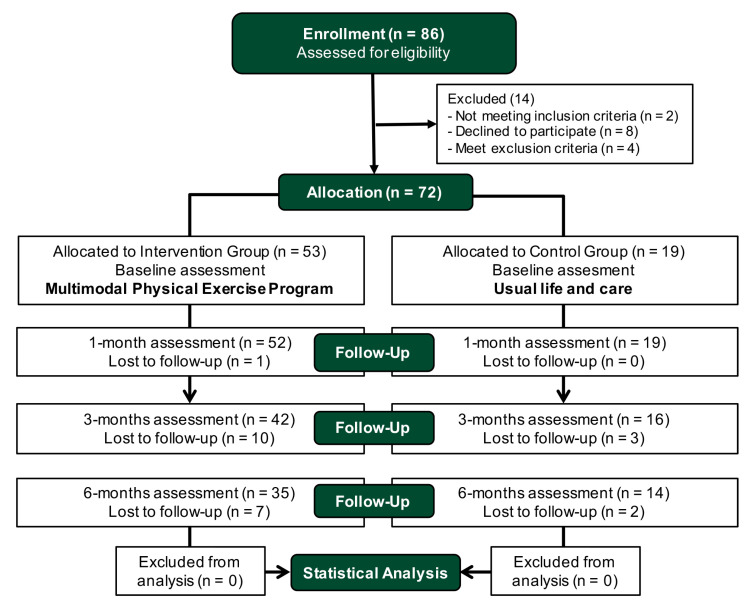
Consolidated Standards of Reporting Trials (CONSORT) flow diagram.

Patients were recruited consecutively from those who requested admission to the SRC-Alzheimer, and were included in the study after selection criteria and voluntarily signing the informed consent (14 patients were excluded in the enrollment; Figure 1). Patients and/or their legal guardian received oral and written information and, after signing the informed consent (by the patient or the legal guardian), the patients were asked to perform the baseline assessment with a multidisciplinary evaluator team. Finally, 72 patients were included who attended the baseline assessment (49 women (68.1%) and 23 men (31.9%)), and met the inclusion criteria: subjects over 50 years of age and diagnosis of AD, with mild or moderate cognitive impairment assessed, scoring between 11 and 23 points on the Mini-Mental State Examination (MMSE) [69], and that signed the informed consent. Exclusion criteria were: (1) other associated pathologies that do not allow physical exercise due to having severe functional disability or being insecure (neurological diseases, severe cardio-respiratory pathology, etc.); (2) impossibility to complete assessment tests due to severe cognitive or physical impairment; (3) falls and other incidents with serious consequences that have caused disability; (4) participation in another intervention program outside of this study, especially an exercise program (important intervention bias for the control group). As withdrawal criteria, we considered: (1) attendance at the MPEP of less than 75% of the total number sessions between each assessment or more than 6 consecutive absences in the intervention group; (2) being admitted to the SRC-Alzheimer or starting a physical exercise program in the control group; (3) any situation that occurs once the study has begun and that involves a significant violation of the study protocol for both groups (e.g., exclusion criteria 4 and 5). 

### 2.4. Masking and Allocation

In this single-blinded study, the assessors were blinded in baseline and follow-up assessments. A multidisciplinary health team, with a neurologist, a neuropsychologist, and a physiotherapist, performed all evaluations without knowing the group to which the patients would be assigned after the baseline assessment. They had no communication with the physiotherapist who applied the intervention during the study. The allocation was performing in cooperation with the Direction Team of SRC-Alzheimer, to decide who was admitted to the center. If at the time of application admission there was a place available and the subject met the other requirements of the SRC-Alzheimer, the subject was admitted and assigned to the intervention group (after selection criteria). If there was no place, the subject was placed on the waiting list and assigned to the control group. An independent researcher, who did not know the identification of the groups, conducted the statistical analysis.

### 2.5. Outcome Measures

In the baseline assessment, all variables were measured including the sociodemographic variables and the cognitive status using the MMSE [69] and the Global Deterioration Scale (GDS) [70]. Later, all outcome variables were measured to assess balance, gait, and bone health status at one, three, and six months. In addition, during the study, all falls of subjects in both groups were recorded.

#### 2.5.1. Balance and Gait Assessment

POMA-T, TUG, OLB, and FR were used to assess balance and gait in the patients. In each test, two trials were conducted and the best score was considered. All these tests are frequently used to assess balance and gait in elderly and frail people and in those with AD or cognitive decline [11,13,22,31,32,33,34,35].

##### Performance-Oriented Mobility Assessment (POMA)

The original 28-point POMA-T version was used. It contains two tests: the POMA-Balance scale (POMA-B) and the POMA-Gait scale (POMA-G). POMA-B assesses sitting balance, get up and sit up from a chair, immediate standing balance in the first 3–5 s, standing balance, balance with eyes closed, and turning balance 360°. The maximum score is 16 points. POMA-G assesses initiation of gait, step height, step length, step symmetry, step continuity, path deviation, trunk stability, and walk stance, and the maximum score is 12 points. In this test, higher scores indicate better performance. Scores less than 19 points indicate a high risk of falling. Between 19 and 24 points, there is a risk of falling, and a score above 24 points indicates there is no disturbance in balance or gait. For this reason, 24 points is considered the cut-off point for predicting falls [11,14,71,72].

##### Timed Up and Go Test (TUG)

The TUG was applied, which measures the time in seconds for the subjects to get up from a standard armchair, walk 3 m, turn, walk back to the chair and sit down. In this test, lower scores indicate better performance. The cut-off point for normal mobility is 12 s and values >30 s indicate a high level of dependence with risk of falling [31,73,74].

##### One-Leg Balance Test (OLB)

The OLB was applied to assess the static balance because it measures the ability of the subject to remain upright on one leg without support for at least 5 s. The test was performed with eyes open, barefoot, using whichever leg was spontaneously chosen by the participant, and each leg was tested. A shorter duration is associated with a twofold increase in the risk of experiencing injurious falls [31,33,75]. An abnormal OLB is considered an independent predictor of cognitive decline in AD [33].

##### Functional Reach Test (FR)

The FR was applied to assess proactive balance; it has a strong association with physical frailty [35,76]. The FR is reliable and valid and measures the distance that the subject is able to reach forward from an initial upright posture to the maximal anterior leaning posture without moving the feet. In this test, higher scores indicate better performance. The cut-off point to determine a risk of falling in older adults is 25.5 cm. Between 25 and 15 cm, the likelihood of a fall is doubled, and those with a score below 15 cm are up to 4 times more likely to fall [35].

#### 2.5.2. Bone Health Status

##### Calcaneal Quantitative Ultrasound (QUS)

The bone health status was assessed using ultrasound calcaneus densitometry/sonometry. Bone mass was measured at the calcaneus (95% of trabecular bone) using quantitative ultrasound (QUS) (Sahara Hologic^®^ Clinical Bone Sonometer; Hologic lnc., Waltham, MA, USA). QUS is a noninvasive method for estimating bone mineral status of the peripheral skeleton. Dual-energy X-ray absorptiometry (DEXA) is currently considered the gold standard and comprises an accurate technique used to measure BMD at specific fracture-related sites, although QUS has been considered a valid and useful measure for determining bone health status in older people [77,78,79]. In addition to bone density, QUS provides some structural information that may be important in determining fracture risk [41] and identifying patients at risk of developing osteoporosis. QUS offers wider accessibility because it is portable, easier to handle, lower in cost, and does not emit ionizing radiation [41,77,78,79].

We measured several parameters commonly generated by QUS to assess the bone health status, such as the T-score (comparison of the average mineral density of the patient’s bone with healthy young people), the estimated BMD (g/cm^2^), and others used as indicators to determine the bone mineral density, such as the speed of sound (SOS; m/s; speed of the ultrasound conduction signal when crossing the calcaneus), the broadband ultrasound attenuation (BUA; dB/MHz; attenuation of broadband ultrasound when crossing the calcaneus), and the quantitative ultrasound index (QUI; expressed as absolute values; QUI = (0.41x SOS) + (0.41x BUA) − 571).

#### 2.5.3. Falls

For our study, a fall was defined as an event in which an older adult unintentionally came to rest on the ground or other lower supporting surface, unrelated to a medical incident or to an overwhelming external physical force [80]. The physical therapist of the SRC-Alzheimer collected the data regarding the falls suffered by the participants of both groups, calling the participants or their legal guardian every month in the control group, and consulting the data recorded by the SRC-Alzheimer health team in the intervention group.

### 2.6. Interventions

The participants in each group had a different place of residence during the study, so that the subjects in the IG were admitted to live in the SRC-Alzheimer, which offers the MPEP, while CG subjects resided in independent living.

The patients assigned to the intervention group were distributed in small groups of 5–8 subjects, maintaining the homogeneity of these groups according to the functional and cognitive baseline status. They carried out an MPEP for 6 months with 3 sessions per week, lasting approximately 45–50 min each. The exercise program developed in small groups provided the benefits of a collective approach and simultaneously allowed the physiotherapist to safely supervise and instruct each patient at all times, even with individualized help from the physiotherapist if necessary in some exercises, such as those of balance.

Patients assigned to the control group were placed on the waiting list for admission to SRC-Alzheimer and continued to live at their usual residence. They did not perform a supervised standardized exercise program. As they were not admitted to the SRC-Alzheimer at that time, the patients and/or their legal guardians, if necessary, were informed that they should continue with their usual care in their daily lives.

#### Multimodal Physical Exercise Program (MPEP)

**Figure 2:**
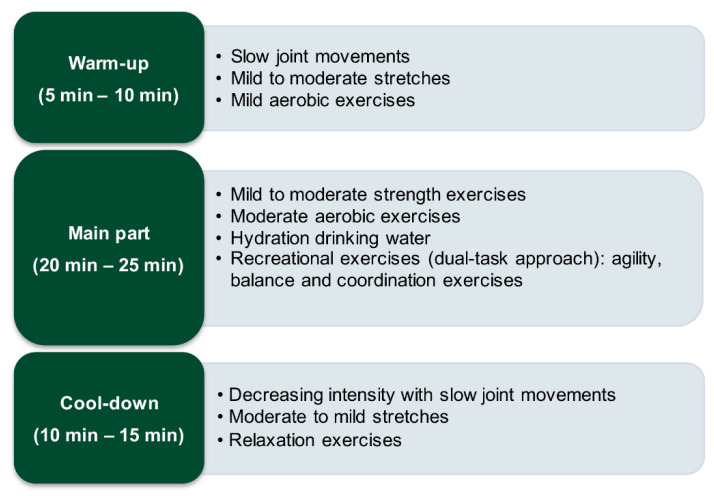
Structure of sessions in the multimodal physical exercise program (MPEP).

The structure of a typical session consisted of 3 well-defined parts [45]: an initial warm-up, the main part, and a cool-down. The main part was usually divided into two parts with a small break in between where patients were invited to hydrate themselves by drinking some water. Session structure is shown in Figure 2.

For all exercises in the program, especially for the aerobic and strength exercises, during the first sessions, the physiotherapist adapted the program to the functional and cognitive states of the group. As such, sessions were carried out following a progression in the dose, intensity, and difficulty of the exercises.

In the warm-up, patients started with simple mobility and stretching exercises, either sitting in a chair or standing, depending on their functional status. Then, they continued with mild aerobic exercises where they combined upper limb movements, pedaling while sitting, walking, etc.

In the main part, the patients performed adapted strength exercises and addressed the main muscle groups, alternating work on the upper and lower quadrant muscles. They performed moderate aerobic exercises combining pedaling while sitting, walking, running, indoor cycling, etc. Dual-task work was included, combining physical exercises with cognitive activities [16,81]. The dual tasks were fundamentally developed in the aerobic exercises and in the recreational part, where coordination, agility, and balance were worked on. It was usually conducted through games with colors, numerical games, word games, right–left laterality, memory games, etc. [16]. The physiotherapist provided instruction on how to complete each exercise, normally performing the exercises before and simultaneously with the patients to help them complete the exercises through imitation.

Finally, during the cool-down, to progressively decrease cardiopulmonary and muscular activity, mild aerobic exercises were performed, and at the end of the session, preferably in a sitting position, patients performed relaxation exercises, trying to control breathing.

### 2.7. Statistical Analysis

Statistical analysis was performed using IBM SPSS Statistics (v.23, IBM Corp., New York, NY, USA). Descriptive analysis of socio-demographic and baseline clinical characteristics was performed, including the frequency of categorical variables and means with standard deviation for quantitative variables. Baseline data were analyzed to calculate differences between groups using ANOVA for quantitative variables and chi-squared for qualitative variables. The variables that showed significant differences between groups in the initial evaluation were considered as a covariate in subsequent analyses. For the dependent variables, we took the estimated means rather than the descriptive means because they are corrected means controlling for the effect of the significant covariates included in the analysis.

Multivariate analysis of variance (MANOVA) was applied with repeated measures (4 levels) to assess interactions between factors, considering time, with the four assessments during the study (baseline, and 1, 3, and 6 months), qualitative grouping variables, covariates, and adding pair-wise comparisons with a post hoc Šidák test, to determine if there were significant differences in the interactions between or within factors. We considered the following as grouping variables: group and sex. For analysis of the falls, we calculated the prevalence of falls during the study, as well as the individual risk (cumulative incidence) and relative risk by group and sex. To analyze whether the values of two or more quantitative variables changed in conjunction, we performed a factorial analysis using principal components to decrease the dimensionality of the data according to the correlations between variables, and then analyzed the correlations of the established components with the remaining variables by correlations with Pearson’s *r* coefficient. The results are expressed as the percentage of shared variance between the variables.

The level of significance for the statistical tests was set to *p* ≤ 0.05 with a 95% confidence interval. Finally, to assess the magnitude of the change in the variables, the effect size of the MPEP was calculated as the partial eta squared (ƞ^2^p) when significant, considering 0.01 as small, 0.06 as medium, and more than 0.14 as a large effect size [82].

## 3. Results

### 3.1. Descriptive Analysis 

We found significant differences in age and cognitive status variables (MMSE and GDS) between groups in the baseline assessment, so these were considered as covariates in subsequent analysis. The other independent variables, including sex, weight, height, and body mass index (BMI), did not present significant differences between the intervention and control group at baseline. Table 1 shows the independent baseline data of each group and their comparison using ANOVA.

**Figure 3:**
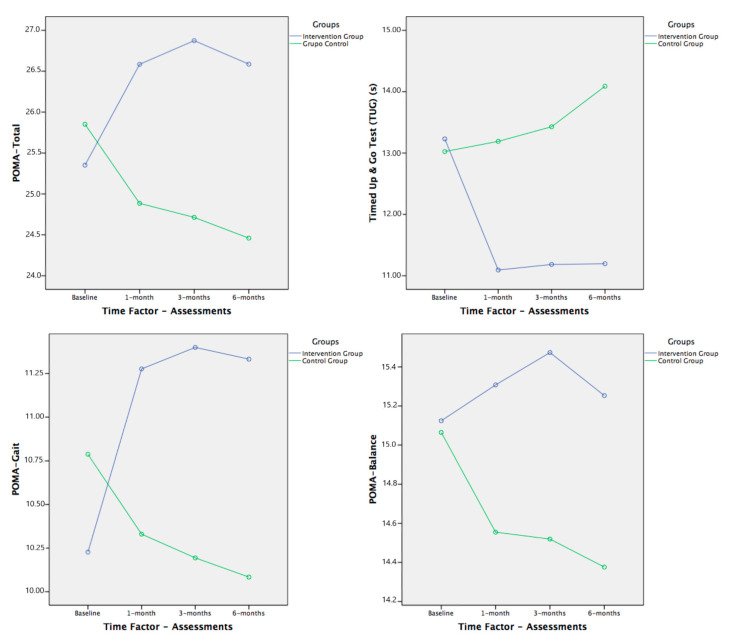
Within-group behavior and follow-up of performance-oriented mobility assessment (POMA) tests and timed up and go (TUG) test. Comparisons between both groups (significant models in multivariate ANOVA (MANOVA) analysis for POMA and TUG tests shown in Table 2 and Table 3).

**Figure 4:**
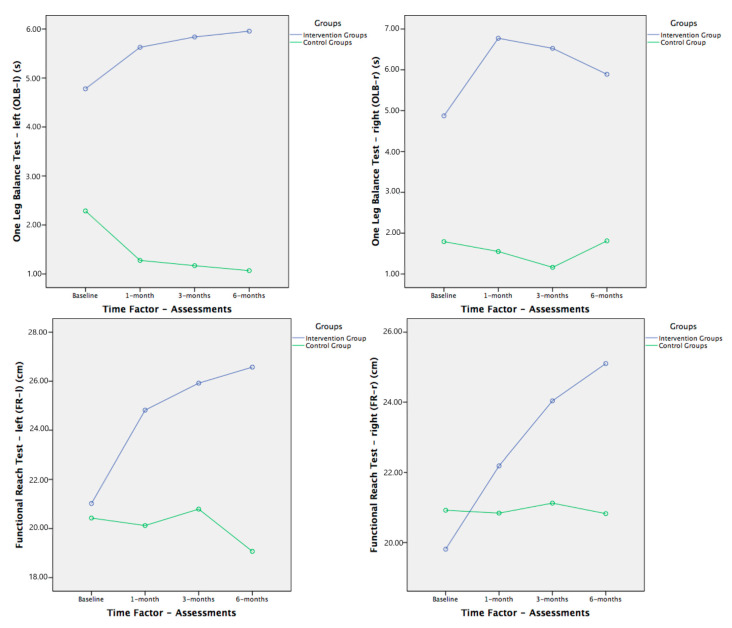
Within-group behavior and follow-up of one-leg balance (OLB) and functional reach (FR) tests. Comparisons between both groups (significant models in MANOVA analysis for OLB test shown in Table 2 and Table 3, and no significant models in MANOVA analysis for FR).

**Figure 5:**
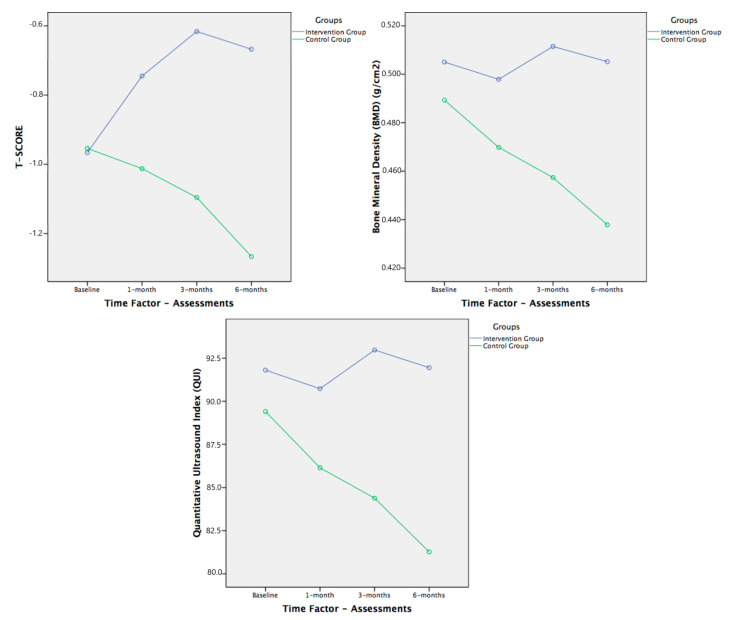
Within-group behavior and follow-up of T-score, bone mineral density (BMD), and quantitative ultrasound index (QUI). Comparisons between both groups (no significant models in MANOVA analysis).

There were no differences between the intervention and control groups at baseline for all physical functions and bone health status outcome measures (ANOVA; *p* > 0.05). We also did not find any differences between sexes, as a unique factor, in any variable. Table 2 shows the changes in the primary and secondary outcomes during the study, reporting considerable improvement in the patients in the intervention group (Figure 3, Figure 4 and Figure 5).

### 3.2. Primary and Secondary Outcomes 

In the inferential analysis with MANOVA, we considered as grouping variables group and sex. In addition, we considered the following as covariates in the outcomes with significant differences between groups in the baseline assessment (Table 1): age, MMSE, and GDS.

The results obtained with the primary outcome variables showed a significant improvement in balance and gait, with a medium to large effect size, in the subjects that completed the MPEP with respect to the control group (Table 3 and Table 4, Figure 3). This improvement was not equal in all variables studied. Especially important was the change in gait, assessed with POMA-G and TUG, both with significant differences in the time × group interaction, where the greatest improvement occurred in the first month of the study, which was maintained until six months. In the control group, the data worsened compared with the beginning of the study.

In terms of balance, as stated above, significant results were obtained in the multivariate analysis for the group factor, either independently or with significant interactions with time or sex factors. That is, we found significant differences between groups regardless of the evaluation and sex (Table 3 and Table 4), except for the FR test, which had no significant differences. However, by observing the trends in all the balance variables in the graphs (Figure 3 and Figure 4), they all improved in the intervention group compared with the baseline assessment, whereas they worsened or remained the same in the subjects in the control group throughout the study.

The results for the state of bone health variables showed no significant differences in any of the possible interactions using MANOVA (Table 3). However, as in the FR, the trends in the results over the six months of the study showed that the results obtained can be considered good (Figure 5), although with aspects to be considered in future studies.

The effect size achieved with the MPEP in the intervention group over time, in comparison with the control group, showed a medium to large effect in the significant interactions. We obtained a more important effect size, as suggested above, for the gait outcomes assessed, POMA-G, TUG, and POMA-T, with large effect (ƞ^2^p = 0.185 to 0.201; Table 3).

To conduct a more detailed analysis of the significant interaction between factors, we applied the post hoc Šidák test to complete pair-wise comparisons and determine which pairs of means had significant differences (Table 4).

### 3.3. Falls

During the study, data were collected regarding the falls suffered by both the subjects of the intervention group, admitted to SRC-Alzheimer, and the subjects in the control group. In these six months of study, 16 subjects had one or more falls, eight in each group (IG = 8; CG = 8; total = 16), and 11 falls were registered in each group (IG = 11; CG = 11; total = 22). There were five multi-fallers, one subject fell three times (IG), four subjects fell twice (IG = 1; CG = 3), and the rest fell only once.

The prevalence at the end of the study (subjects who fell) was 15.09% in the intervention group and 42.11% in the control group, showing statistically significant differences between the groups (χ^2^ = 5.904; *p* = 0.015). However, when comparing the number of falls between the groups, no significant differences were found between the groups (*p* = 0.065) after the correction in degrees of freedom with the Welch test because there were significant differences in Levene’s contrast test since the highest variance was in the lowest group (CG). We found no significant differences by group and sex in falls. In the intervention group, the prevalence was 21.1% in men and 11.8% in women (χ^2^ = 0.820; *p* = 0.365). In the control group, the prevalence was 25.0% in men and 46.7% in women (χ^2^ = 0.608; *p* = 0.435).

With these results, the cumulative incidence or individual risk of a fall in the subjects in the intervention group was 15.1%, and 42.1% in the subjects in the control group. The relative risk (RR) of the intervention group with respect to the control group was 0.36 (95% CI: 0.16–0.82). With an RR much lower than one, there was an association between participating in the MPEP and suffering a fall, with a protective intervention against the risk of suffering a fall. Again, we found no significant differences by sex either in individual risk (χ^2^ = 0.005; *p* = 0.946) or in relative risk (RR = 1.01 (95% CI: 0.78–1.32)).

### 3.4. Correlations between Variables

The factorial analysis initially showed that by applying principal components analysis, three components of measurement sets clearly formed that were independent of each other. The same occurred for each evaluation of the study: (1) Component-1 (bone densitometry), the five variables of bone densitometry (T-score, BMD, QUI, BUA, and SOS); (2) Component-2 (OLB and FR), the four variables of the OLB and FR tests (OLB-r, OLB-l, FR-r, and FR-l); and (3) Component-3 (POMA), the three variables of POMA (POMA-T, POMA-B, and POMA-G).

These three principal components always explained more than 84% of the accumulated variance over the total explained variance of the components in each evaluation: Component-1 explained 40.1–49.0%, Component-2 explained 21.5–30.7%, and Component-3 explained 13.4–16.3%. For this reason, we used the three components as new variables to calculate the correlations with the TUG, falls, and the rest of the independent variables that were not included in these components.

When calculating the correlations between variables, we found several significant associations, but, as indicated above, to look for the most powerful correlations, we express these correlations as percentages of shared variance. Thus, in the analysis, several significant correlations between outcome variables were identified, in some cases with high percentages of shared variance between variables, although most of them were in men from the control group. Considering the small number of men in the control group, we must consider these data with caution. Therefore, we can only consider the significant correlations (*p* < 0.001) between the TUG and POMA as relevant with a shared variance between 32.5% and 37.6% in the different evaluations and independent of the group and sex.

## 4. Discussion

In this study, we analyzed the effects of a six-month MPEP in institutionalized older people in a State Reference Center for people diagnosed with Alzheimer’s disease, compared to community-dwelling older people with Alzheimer’s disease. Considering the frailty of this population and the comorbidities associated with aging and cognitive impairment, we analyzed the effects of MPEP on several outcome variables, including physical functions, especially gait and balance, and the state of bone health. Improving physical conditions can lead to a reduction in the risk of falls and bone fractures, as well as to their subsequent serious consequences. These benefits can lead to a better quality of life and a greater degree of autonomy for these patients. With our results, we contribute to the evidence on interventions aimed at reducing falls, bone fractures, and their consequences in older people with AD [2,16,31,32,42,44]. In the design, and during the study development, we considered some possible differences between both groups, fundamentally those derived from the fact that their place of residence was different, and that we were comparing institutionalized with community-dwelling older people with Alzheimer’s disease. Some of these differences were considered in the selection and/or withdrawal criteria, but others were not included in the statistical analysis due to the difficulty of being able to be controlled, and for that reason it is included as a limitation of the study to be considered in future trials.

In terms of our initial objectives and hypotheses, an MPEP in institutionalized patients with AD had beneficial and significant effects on gait and balance functions, improving both over the course of the study, fundamentally in the first month of intervention. The greatest effect was achieved in the evaluation one month after the beginning of the intervention. This suggests that starting activity in older persons with AD through supervised multimodal exercise programs obtains results quickly. However, the results over six months also suggest that this type of physical exercise should be maintained in order to continue improving, maintaining, and/or not allowing the loss of capacities, characteristic of aging and frailty, to continue. We identified a clinically relevant effect on the state of bone health, although without statistically significant differences, possibly due to the duration of the study and the size of the sample. These results are consistent with many previous studies in which exercise was a beneficial intervention in older people with AD or cognitive impairment.

### 4.1. Intervention

Exercise is a safe method of improving physical performance and relieving the symptoms of various diseases. In our study, we considered the beneficial effects of exercise as a preventive or therapeutic intervention in AD and bone health [83]. We also considered the beneficial effects of physical exercise on risk factors for falls and fractures, such as cognitive decline, bone mass loss, gait, and balance, as well as the positive effects on quality of life and autonomy in ADLs for AD patients, as stated in some previous studies [2,32,84].

Exercise is one of the most common elements of both multifactorial and multiple component interventions and is an effective single intervention for preventing falls [85]. Many studies reported that the best intervention is one that covers different aspects, such as strength, flexibility, balance, agility, coordination, etc. [10,83,86], and physical as well as cognitive and other aspects [7,16,81,87,88,89]. However, the results about which exercise programs are best related to fall risk prevention are inconclusive [90].

The MPEP that we applied in this study proved to be a safe and positive intervention in relation to the objectives set out from the beginning of the study. The combination of different types of physical exercises, such as aerobic, strength, coordination, agility, etc. [91], in combination with a dual task aimed at cognitive functioning, is in line with previous studies [16,81]. In addition, we conducted a group intervention to enhance the beneficial effects of collective work, although small enough (five to eight participants) [16] to provide individualized attention according to the needs of each patient [91]. Despite the American College of Sports Medicine and some other studies providing evidence of the beneficial effects of unsupervised exercise [41,48,92], other studies reported a greater effect produced by systematized and supervised exercise [31,91,93]. This appears to be more important in people with AD or any cognitive impairment [16]. For us, the supervised work in small groups was fundamental to providing the exercises simultaneously, allowing the subjects to complete the activities by imitation, mainly in those with greater cognitive deterioration.

Some similar studies lasted between seven weeks and one year, where studies of one year, and especially studies of six and three to four months, are popular [90,94]. Therefore, our study could be considered a medium-duration study. Most studies propose a frequency of two to three weekly sessions, and the durations of the sessions varied greatly, between 20 and 75 min, although most of the studies referenced in this manuscript provided sessions between 45 and 60 min [10,45,90,94,95]. Exercise types and doses are dependent on participants’ adherence to the intervention, with the variability in previous studies making it difficult to obtain conclusive results [10,41,45,85,86,90,91].

In summary, our intervention was well designed, in line with the evidence, and was conducted and adapted to the specific characteristics of each patient in each intervention subgroup.

### 4.2. Effects on Falls

Intrinsic fall-related risk factors include advanced age, female sex, osteoporosis, reduced vision, impaired balance and gait, muscle weakness, polypharmacy, history of previous falls, chronic diseases, and impaired cognitive status, especially attention and executive dysfunction [85,91,96]. We considered assessing some of them, such as falls, balance and gait disturbances, and bone health status, to analyze the effects of an MPEP designed to reduce the risk of falls and fractures in patients with AD.

Fall prevention interventions that address two or more risk factors with multifactorial approaches significantly further reduce the risks of falls in older people [85]. However, not all studies reported these same positive effects [97].

In people with AD, the associations of possible risk factors with falls were studied more than interventions for the reduction of their prevalence [98,99,100]. Physical exercise clearly appears to be a protector against falls not only in older people but also in people with AD and other dementias [101,102]. In older people, the prevalence of falls is around 33%, as reported in most of the literature [103,104,105,106]. As mentioned above, the prevalence of falls in people with dementias or AD in particular is twice as high, that is, the fall prevalence is between 60% and 80% [107]. In our study, the sample prevalence of falls at the end of the study (six months) was 22.22% (IG = 15.1%; CG = 42.1%) (correcting to one year would be 44.44%), which is below the general value. Another study reported a prevalence of 51.4% in people with AD and 33.3% in the control group [101]. Our data, with a prevalence in the intervention group of 15.09% at the end of the study, were more similar to those reported by Eshkoor et al., with a prevalence of 17% in six months, which was higher in women than in men [108]. The calculation of relative risk (RR = 0.36) indicated that the MPEP is a protective intervention against falls in institutionalized AD patients, in line with reports from other studies [101,102].

The few falls recorded in the intervention group throughout our study were due in part to baseline physical condition, the tests used to assess balance and gait indicated a low risk of falls in our subjects, and in part to the effectiveness of the intervention. This may have led to the low sensitivity in the functional tests used in our trial as predictor tests of falls. The environmental conditions at SRC-Alzheimer, a State Reference Center with the best facilities, qualified professionals, and the best possible ratio caregiver/patient of centers for people with AD and other dementias, may also have contributed.

### 4.3. Effects on Gait and Balance

To study the effect of the intervention on physical functions, balance and gait disturbances were evaluated as risk factors for falls. Some of the most commonly used tests in elderly people and Alzheimer’s patients were used for this purpose. However, the variety of parameters around the interventions and assessment tools used in the previous studies complicates comparisons. Scientific and clinical consensus on these aspects is required.

The data obtained showed a significant improvement in our primary outcomes, especially on gait. In the POMA-T, POMA-G, and TUG, despite the three tests already showing a low risk of falls from the beginning in our sample, we found significant differences between and within groups (time × group interaction) with a large effect size (ƞ^2^p = 0.185 to 0.201). In the POMA-T, the risk of falling begins to appear below 24 points; in the POMA-G, the risk of falls is below seven points [101]; and in the TUG, a low risk of falls is indicated between 10 and 20 s [73,108,109]. In our study, baseline values of POMA-T and POMA-G were above 25.5 points and 10 points, respectively, and just over 13 s in the TUG.

In these tests, the most improvement occurred in the first month of intervention and was maintained afterward, which showed that starting to lead a more active life with physical exercise in a systematic supervised program produces an improvement in gait [110,111,112].

These results are consistent with other studies that provided physical exercise programs, which also reported positive effects with significant differences in POMA-T, even much higher than ours, as in the study by Mirolsky-Scala et al. [113], increasing from 8 to 16 points. In our study, the intervention group went from 25.21 to 26.29 points. This difference may be due to the subjects in our study starting from a much higher score, which would be harder to improve, as the maximum score is 28 points. Values similar to those in our study were reported with a 12-week intervention [2]. Our subjects had very high starting scores, and in spite of that, they managed to improve the physical function by means of the POMA. Sterke et al. [114] presented baseline data of 18.7 points overall, 9.2 points in balance, and 8.7 in gait, and Bossers et al. [115] presented an average of 8.5 points for the POMA test in people with dementia in their review.

For the TUG, an improvement was observed in our sample with the intervention, reducing the result of the test by two seconds, and can be compared with other studies. De Andrade et al. [116] and Yao et al. [117] reported reductions of about two seconds with a four-month intervention, an improvement that we already obtained in the first month, which was maintained.

Regarding balance, our results were not as significant. Given the trends shown by both groups being similar to the gait tests, we can consider them positive and clinically relevant.

In the POMA-B and OLB tests, we only found significant differences between groups, regardless of the duration of the intervention with a medium effect size (ƞ^2^p = 0.091 to 0.104), and since there were no differences between groups at the beginning of the study, we can assume the effect of the intervention due to subsequent evaluations.

The same occurred in the FR as in the gait tests, since our sample already presented higher values at the initial evaluation than those described by Duncan and Weiner [35,76] for the higher risk of falls, placing our subjects at low risk with approximate values of 21 cm.

Our results showed that our sample, according to Duncan [118], is twice as likely to fall, regardless of the role played by AD. These values are in agreement with those described by Brauer [119] 20.8. ± 8.13 cm) and Newton [120] (22.6 ± 8.64 cm) in the anterior reach, as this sample included healthy older people. In relation to works with people with AD and other dementias, our values are well above any other study [121], but, in general, they are in accordance with the bibliography.

Our intervention group improved a little more than 4 cm with the right arm and a little more than 2 cm with the left, which is consistent with the results presented by Miu et al. [122]. They reported a 3 cm improvement with three months of intervention, although the improvement was somewhat less in AD than in vascular dementia. According to results published by Vreugdenhil et al. [123], a 3 cm improvement was attained in four months of intervention.

Contrary to previous descriptions, in the OLB test, our sample produced results below five seconds (approximately 4.5 s), which indicated a clear risk of falls. Our data are consistent with those presented by Bossers [115] and Rolland [32] in their systematic reviews, where they showed that more than 90% of people completed the test faster than five seconds, but in a sample with subjects with higher cognitive impairment (MMSE = 8.8) than our subjects.

If compared with the results of studies in older people without cognitive impairment, our results are far below, which shows that cognitive impairment has a significant influence on balance. In Briggs et al. [124], with a sample of 71 subjects aged 72.25 years, the average was 20.43 s with the dominant lower limb and 19.94 s with the non-dominant one. According to the results of Bohannon et al. [125] in subjects between 60 and 69 years, the mean was 22.5 s, and in subjects between 70 and 79 years, the mean was 14.2 s. The test was performed in both cases with open eyes.

The results were less satisfactory in this test, perhaps because the static balance, with such a low support base, is more affected by the disorders associated with cognitive impairment in AD.

In our study, the effects were evident from the first month of intervention and remained up to six months, which differs from another study that reported that the main adaptations to exercise occur from months three to four and that the greatest changes occur after six months of training [126]. We believe that perhaps it is necessary to open lines of research to assess the type of interventions that have a faster effect on improving indicators of physical frailty in older people with and without AD.

### 4.4. Effects on Bone Health Status

Osteoporosis is widely related to falls and fractures, as is AD [96,127], and many authors have focused their research on evidence of comorbidity between AD and osteoporosis or loss of BMD using a cross-sectional view [29,128,129]. There is practically no evidence of the efficacy of some type of exercise intervention on BMD in AD. Clinical trials aimed at the effects of exercise in older people or postmenopausal women are more frequent [86,130,131,132,133,134]. Although the evidence appears to support the assumption that exercise has positive effects on bone status, not all studies provided clear evidence of increased BMD with exercise interventions [97].

Our baseline data differ quantitatively from reports by other authors, since the BMD obtained in our sample was 0.506 ± 0.150 g/cm^2^, whereas the data from other authors in patients with AD ranged from 1.110 to 2.124 g/cm^2^ [128,135,136,137,138]. This difference may be due to others using values obtained by DEXA in the neck of the femur and we used calcaneal QUS. The T-score in our study places our subjects on the limit of osteopenia (T-score = −1.06 ± 1.09), similar to the data on a population of 440 older subjects with an average age of 80 years (T-score = −0.99), where they obtained values using DEXA in the left trochanter [139]. Castrillón et al. [140] reported a T-score below −1.6 using DEXA in the calcaneus, although there is no consensus among the authors [141]. 

In our study, the values of the variables of the calcaneal bone densitometry in the patients in the intervention group were maintained or even increased slightly, while the values in the control group worsened throughout the six-month study. The BMD remained more or less constant in the intervention group, while the BMD in the control group progressively decreased to 12.25%. In the T-score, the values in the intervention group increased by 24.5% at six months, no longer within the limits of osteopenia and reaching the range of normality in terms of bone health status (T-score = 0.80 ± 1.26). 

Exposure to sunlight, with or without associated pharmacologic treatment, produced significant and beneficial effects on BMD in patients with AD in a review of randomized clinical trials, where metacarpal BMD increased by 2.3–4.1% in the intervention groups and decreased by 0.9–5.6% in the control groups [142]. The incidence of fall fractures also decreased in the intervention groups. Therefore, physical exercise should be added to other interventions, which would further improve the parameters of bone densitometry while reducing the risk of falls, with special attention on strength exercises to work on the muscle–bone relationship [132]

A review stated that short-term moderate-intensity aerobic exercise and long-term high-intensity resistance exercise can prevent osteoporosis and improve balance, helping to prevent falls and fractures [83]. The potential causal association between physical activity and osteoporotic fractures should be considered from an epidemiological viewpoint [143].

However, more studies on the effects of the different interventions are necessary, including physical exercise. The current evidence on bone loss in AD is insufficient, and this merits critical attention because this work could uncover novel diagnostic and therapeutic opportunities needed to address AD [29].

### 4.5. Strengths and Limitations

The strengths of this study are related to the procedures carefully conducted by a multidisciplinary professional group highly qualified in the care and treatment of patients with AD. This allowed the evaluations to be completed, including a supervised MPEP in small groups, with a high level of individualization for the exercises. One of the main strengths of our study is the amount and variety of tests used to evaluate physical functions, as well as the collection of data on falls and the assessment of BMD using calcaneal QUS. This allowed us to analyze the effects of the intervention on these outcome variables and the associations and correlations between them. Another strength is the follow-up on the control group, and recruiting older people diagnosed with AD to the control group, who did not perform any type of systematic or supervised physical activity. For this to not bias the study, we ensured that this circumstance did not occur, and not many studies did so. Most studies, when referring to the control group, did not clarify whether they controlled or supervised the subjects to conduct any type of physical activity. 

This study also has several limitations. The sample size was calculated with a 3:1 ratio between the intervention and control groups. Despite having a significant number in the intervention group, conducting further studies with a larger sample in a 1:1 ratio would increase the power of the beneficial results in this study. The initial differences between the groups in the level of cognitive impairment could have conditioned the results, and possibly was produced by the non-randomized assignment of the subjects to the study groups. In the same way, no other possible differences between the groups have been considered and should be studied in future trials, such as the level of activity during the study, diet, particular conditions of dwellings in the control group, more information about how the falls occurred, etc. In future studies, the difficulty in maintaining the sample in similar long-term studies must be considered and the estimate of losses in the calculation of the sample size will have to be increased. Finally, in the future, it would be desirable for other studies to look at if the effect of the program is maintained when participants no longer are part of the exercise program.

Large amounts of evidence interrelate different aspects in AD that were addressed in our clinical trial. Aging and the cognitive deterioration of AD patients, which place these patients in an evident state of frailty, are clearly associated and correlated with the risk of falls, osteoporotic fractures, loss of bone mass, and alterations in walking and balance. Some studies reported some of them as risk factors or predictors of the others. More studies are needed to understand the related underlying physiological mechanisms, as well as the effects of the best possible interventions. Either combined or multicomponent treatments, which seem to be the ones that produce the best results, or programs implemented with some specific task addressed to some certain factor, should be used to assess both the specific and the global effect on the patient.

In future studies, the size and homogeneity of the sample should be increased, subjects should be carefully sub-classified, and consensus should be reached among the tests to be performed, with long-term follow-up of physical exercise programs, alone or in combination with other interventions, which should be described in detail to allow for better replication of the studies. In addition, the physiopathological mechanisms underlying all these alterations in patients with AD and their correlations should be further investigated.

## 5. Conclusions

A multimodal physical exercise program produced positive effects with statistically significant differences between and within groups in the short and medium term, improving balance and gait in institutionalized patients with Alzheimer’s disease. Positive and clinically relevant effects were found, although without significant differences in bone health, slowing bone loss. Some significant, although insufficient, correlations were found between physical tests and BMD parameters in Alzheimer’s patients, as well as with recorded falls, although studies with larger sample sizes should be conducted to increase the validity of these associations and the results in general. These results should be used to further nuance the parameters of exercise programs in study designs for the best possible treatments of Alzheimer’s disease.

## Figures and Tables

**Table 1:** Baseline data of independent variables in the intervention and control groups. The continuous variables are shown as means ± standard deviation, and the categorical variables are shown as % (*n*). To analyze baseline differences by group, we conducted ANOVA or chi-squared tests for quantitative and qualitative variables, respectively, with covariates if significant.

Variable	Intervention Group	Control Group	*p*-Value
Gender, women % (*n*)	64.15 (34)	78.95 (15)	0.235
Age (year)	78.19 ± 9.87	72.79 ± 8.42	0.037 *
Weight (kg)	65.69 ± 13.81	70.42 ± 9.71	0.516
Height (m)	1.56 ± 0.87	1.58 ± 0.96	0.777
BMI (kg/m^2^)	26.97 ± 4.26	28.66 ± 4.98	0.160
MMSE (points)	15.49 ± 5.12	21.42 ± 3.06	<0.001 *
GDS (points)	4.81 ± 0.81	3.89 ± 0567	<0.001 *
Falls (*n*)	0.21 ± 0.57	0.58 ± 0.77	0.065 **

* Indicates significant differences between groups; ** Levene’s contrast test showed significant differences and since the highest variance was found in the lowest group (control group), correction in degrees of freedom was applied using the Welch test. BMI, body mass index; MMSE, Mini-Mental State Examination; GDS, Global Deterioration Scale.

**Table 2:** Changes in primary and secondary outcomes from baseline to 6 months and between-group differences with age as a covariate when significant.

Variable	Gr.	Intervention Group (Estimated Means ± SD)				Between-Group Difference (IG–CG)				
		Baseline	1 Month	3 Months	6 Months	Mean	SEM	*p* Value	95% CI (Lower–Upper)	
**POMA-T (points)**	**IG**	25.21 ± 3.01	26.38 ± 2.20	26.74 ± 2.02	26.29 ± 2.21	1.372	0.769	0.082	−0.180	2.923
	**CG**	26.14 ± 1.56	25.57 ± 1.45	25.07 ± 1.00	24.79 ± 1.58					
**POMA-B (points)**	**IG**	15.06 ± 1.10	15.21 ± 0.91	15.44 ± 0.82	15.12 ± 0.88	0.661	0.296	0.031 *	0.064	1.258
	**CG**	15.14 ± 0.66	14.93 ± 0.73	14.71 ± 0.47	14.57 ± 0.51					
**POMA-G (points)**	**IG**	10.15 ± 2.12	11.18 ± 1.59	11.29 ± 1.40	11.18 ± 1.47	0.710	0.535	0.191	−0.368	1.789
	**CG**	11.00 ± 1.18	10.64 ± 1.08	10.36 ± 0.63	10.21 ± 1.12					
**TUG (s)**	**IG**	13.58 ± 4.85	11.33 ± 2.42	11.56 ± 2.87	11.57 ± 2.97	−1.968	0.854	0.026 *	−3.687	−0.248
	**CG**	13.09 ± 2.74	12.91 ± 2.85	13.18 ± 2.95	14.18 ± 3.23					
**OLB-r (s)**	**IG**	4.58 ± 5.93	5.96 ± 6.87	5.83 ± 7.49	5.13 ± 5.43	4.437	2.064	0.037 *	0.275	8.598
	**CG**	6.23 ± 10.15	6.01 ± 8.14	5.82 ± 7.45	5.36 ± 7.38					
**OLB-l (s)**	**IG**	4.45 ± 5.83	4.84 ± 6.75	5.03 ± 6.66	5.18 ± 5.89	4.099	2.046	0.051	−0.027	8.225
	**CG**	6.72 ± 9.22	5.85 ± 7.73	4.88 ± 6.13	4.60 ± 5.66					
**FR-r (cm)**	**IG**	19.42 ± 9.17	21.80 ± 8.77	23.52 ± 8.03	24.69 ± 7.17	1.857	2.635	0.485	−3.458	7.172
	**CG**	24.20 ± 7.52	24.06 ± 7.01	24.06 ± 6.73	23.63 ± 6.51					
**FR-l (cm)**	**IG**	20.86 ± 8.44	24.31 ± 7.47	25.47 ± 7.61	25.77 ± 7.72	4.481	2.672	0.101	−0.908	9.869
	**CG**	23.60 ± 9.94	23.87 ± 8.89	24.39 ± 7.83	22.94 ± 7.39					
**T-Score**	**IG**	−1.06 ± 1.09	−0.90 ± 1.13	−0.77 ± 1.18	−0.80 ± 1.26	0.333	0.395	0.404	−0.463	1.130
	**CG**	−1.06 ± 0.97	−1.16 ± 0.93	−1.16 ± 0.87	−1.31 ± 0.92					
**BMD (g/cm^2^)**	**IG**	0.49 ± 0.13	0.48 ± 0.13	0.49 ± 0.13	0.49 ± 0.14	0.041	0.046	0.372	−0.051	0.134
	**CG**	0.49 ± 0.11	0.45 ± 0.10	0.45 ± 0.10	0.43 ± 0.10					
**BUA (dB/MHz)**	**IG**	73.29 ± 20.29	73.84 ± 19.34	74.89 ± 20.69	74.61 ± 23.68	8.230	6.600	0.219	−5.072	21.532
	**CG**	74.72 ± 14.84	65.59 ± 13.01	65.08 ± 12.60	67.69 ± 13.50					
**SOS (dB/MHz)**	**IG**	1537.09 ± 31.90	1533.47 ± 31.54	1537.77 ± 31.90	1534.75 ± 36.26	8.321	11.42	0.470	−14.695	31.337
	**CG**	1534.53 ± 27.17	1529.29 ± 25.63	1530.24 ± 26.85	1521.19 ± 27.01					
**QUI**	**IG**	88.91 ± 20.61	87.95 ± 20.05	90.18 ± 20.95	89.57 ± 22.29	6.563	7.249	0.370	−8.047	21.172
	**CG**	89.01 ± 16.95	83.32 ± 16.20	83.02 ± 15.49	80.43 ± 16.10					

* Indicates significant differences between groups, regardless of the evaluation and sex. SD, standard deviation; IG, intervention group; CG, control group; SEM, standard error of the mean; CI: confidence interval; POMA-T, Tinetti’s performance-oriented mobility assessment; POMA-B, balance performance-oriented mobility assessment; POMA-G, gait performance-oriented mobility assessment; TUG, timed up and go test; OLB-r, one-leg balance test—right; OLB-l, one-leg balance test—left; Fr-r, functional reach test—right; FR-l, functional reach test—left; BMD, bone mineral density; BUA: broadband ultrasound attenuation; SOS, speed of sound; QUI: quantitative ultrasound index.

**Table 3:** Inferential analysis used to calculate the interaction between factors using multivariate analysis of variance (MANOVA). Only statistically significant interaction models with the grouping variables and the significant covariates in the model are shown. Effect size is expressed as the partial eta squared (ƞ^2^p).

Variable	Covariate	Grouping Variables			Significant Model			
	Age	Time	Group	Sex	Interaction	MANOVA (F)	*p*-Value *	(ƞ^2^p)
POMA-T	✓	✓	✓	✓	Time * group	3.105	0.037	0.185
POMA-B	✓	✓	✓	✓	group	4.980	0.031	0.104
POMA-G	✓	✓	✓		Time * group	3.526	0.023	0.197
TUG	✓	✓	✓		Time * group	3.606	0.021	0.201
OLB-r	✓	✓	✓	✓	group	4.622	0.037	0.097
OLB-l	✓	✓	✓	✓	Group * sex	4.289	0.044	0.091
FR-r	✓	✓	✓	✓	no significant model	-	-	-
FR-l	✓	✓	✓	✓	no significant model	-	-	-
T-Score		✓	✓	✓	no significant model	-	-	-
BMD		✓	✓	✓	no significant model	-	-	-
BUA		✓	✓	✓	no significant model	-	-	-
SOS		✓	✓	✓	no significant model	-	-	-
QUI		✓	✓	✓	no significant model	-	-	-

* Only significant differences in the interactions are shown. (ƞ p), partial eta squared; POMA-T, Tinetti’s performance-oriented mobility assessment; POMA-B, balance performance-oriented mobility assessment; POMA-G, gait performance-oriented mobility assessment; TUG, timed up and go test; OLB-r, one-leg balance test—right; OLB-l, one-leg balance test—left; Fr-r, functional reach test—right; FR-l, functional reach test—left; BMD, bone mineral density; BUA, broadband ultrasound attenuation; SOS, speed of sound; QUI, quantitative ultrasound index.

**Table 4:** Pair-wise comparisons using Šidák post hoc test (MANOVA) in physical function variables. Only statistically significant differences in the comparisons of the significant MANOVA interaction models are shown.

Variables and Significant Model (MANOVA)				
Grouping Variable	(I)	(J)	Between-Factor Difference (I–J) (95% CI)	*p*-Value
POMA-T (time * group with age)				
IG	Baseline	1 month	−1.233 (−2.108 to −0.358)	0.002
		3 months	−1.522 (−2.553 to −0.491)	0.001
		6 months	−1.235 (−2.271 to −0.198)	0.012
1 month	IG	CG	1.700 (0.034 to 3.366)	0.046
3 months	IG	CG	2.160 (0.629 to 3.692)	0.007
6 months	IG	CG	2.127 (0.468 to 3.785)	0.013
**POMA-B (group with age)**				
Group	IG	CG	0.661 (0.064 to 1.258)	0.031
POMA-G (time * group with age)				
IG	Baseline	1 month	−0.989 (−1.633 to −0.345)	0.001
		3 months	−1.101 (−1.827 to −0.376)	0.001
		6 months	−0.992 (−1.782 to −0.202)	0.007
3 months	IG	CG	1.130 (0.352 to 1.907)	0.005
6 months	IG	CG	1.186 (0.323 to 2.048)	0.008
TUG (time * group with age)				
IG	Baseline	1 month	2.202 (0.758 to 3.646)	0.001
		3 months	2.012 (0.494 to 3.531)	0.004
		6 months	1.960 (0.491 to 3.429)	0.004
1 month	IG	CG	−2.137 (−3.652 to −0.623)	0.007
3 months	IG	CG	−2.321 (−3.997 to −0.646)	0.008
6 months	IG	CG	−3.172 (−5.048 to −1.295)	0.001
**OLB-r (group with age)**				
Group	IG	CG	4.437 (0.275 to 8.598)	0.037
OLB-l (group * sex with age)				
CG	Men	Women	−7.525 (−14.840 to −0.210)	0.044
Men	IG	CG	8.225 (0.860 to 15.589)	0.029

IG, intervention group; CG, control group; POMA-T, Tinetti’s performance-oriented mobility assessment; POMA-B, balance performance-oriented mobility assessment; POMA-G, gait performance-oriented mobility assessment; TUG, timed up and go test; OLB-r, one-leg balance test—right; OLB-l, one-leg balance test—left.

## Data Availability

The data presented in this study are available on reasonable request from the corresponding author. The data are not publicly available due to the applicable data protection law.
